# When Pap Testing Fails to Prevent Cervix Cancer: A Qualitative Study of the Experience of Screened Women Under 50 with Advanced Cervix Cancer in Canada

**DOI:** 10.7759/cureus.3950

**Published:** 2019-01-24

**Authors:** Mark T Corkum, Heather Shaddick, Elizabeth Jewlal, Nikhilesh Patil, Eric Leung, Akira Sugimoto, Jacob McGee, Michel Prefontaine, David D'Souza

**Affiliations:** 1 Radiation Oncology, London Regional Cancer Program, University of Western Ontario, London, CAN; 2 Medical Physics, London Regional Cancer Program, University of Western Ontario, London, CAN; 3 Radiation Oncology, Dalhousie University, Halifax, CAN; 4 Radiation Oncology, Toronto Sunnybrook Hospital, University of Toronto, Toronto, CAN; 5 Oncology, London Regional Cancer Program, University of Western Ontario, London, CAN; 6 Neurology, London Regional Cancer Program, University of Western Ontario, London, CAN

**Keywords:** cervical cancer, pap smear, cancer screening, patient experiences, qualitative research

## Abstract

Introduction: While Papanicolaou (Pap) smears have resulted in a significant decline in cervical cancer incidence and mortality, our clinical experience indicates some women still present with locally advanced cervical cancer (LACC) despite having received Pap smear screening. Recent guidelines have decreased the recommended frequency of Pap smears to every three years. Our study sought to investigate the experiences of young women compliant with cervical screening who presented with LACC.

Methods: Women under 50 with LACC, FIGO (International Federation of Gynecology and Obstetrics) stage IB1 to IVA who underwent a Pap smear within two years of diagnosis and received curative intent chemoradiotherapy between September 2010 and December 2012 were included. Participants were treated at a tertiary academic cancer centre and invited for a semi-structured, in-person interview, which was analysed qualitatively using thematic analysis.

Results: Thirteen out of 38 women had Pap screening two or less years before diagnosis. Ten consented to participate in an interview. Several key themes emerged: I) Belief that LACC does not occur in those who undergo screening; II) Lack of understanding about LACC symptoms/diagnosis of cervix cancer; III) Reluctance from health care providers to perform a detailed pelvic examination in the presence of symptoms; IV) Negative emotions including anger, shame, regret, mistrust; V) Changes in quality of life from treatment; VI) Advice for other women.

Conclusions: One-third of women presenting with LACC had appropriate Pap screening prior to diagnosis. Patients believe delays in their diagnosis resulted in detrimental quality of life. There is a need to educate physicians and the public about the symptoms of cervix cancer and to consider this diagnosis even when Pap screening has occurred.

## Introduction

Although cancer of the cervix remains a leading cause of death in women worldwide, in developed countries, the death rate has decreased significantly from the 1960s with the introduction of Papanicolaou (Pap) smear screening [1]. This is due to the implementation of organized screening programs and the development of national and provincial consensus guidelines. The Canadian Task Force on Preventative Health Care states that “most advanced cervical cancer (and associated mortality) occurs among women who have never undergone screening or who have had a long interval between Pap tests” [2]. In accordance, the Canadian Task Force has decreased the frequency of routine screening to every three years with women beginning screening at age 25. This is consistent with other international cervical cancer screening guidelines [3-4]. As a result, there may be a perception that cervix cancer is unlikely in a woman compliant with screening. In our population, women underwent Pap smears alone without human papillomavirus (HPV) co-testing, in most cases through their primary care physician.

Previous studies have demonstrated that Pap smears are not perfect, and that screen failures can occur. In the United Kingdom, one study of 66 patients with newly diagnosed invasive cervix cancer found 30 screen failures with 18 (27%) having a negative smear in the preceding five years [5]. In an audit of screening failures in 246 women in Calgary, Canada, a number of factors were identified including no screening, underscreening, undersampling, delay in referral for abnormal Pap smear, or underdiagnosis on colposcopy [6].

Our clinical experience suggested there was a proportion of women who present with locally advanced cervical cancer (LACC) who received recommended Pap smear screening prior to diagnosis, despite the negative predictive value of developing an in-situ or malignant cervical cancer of over 99.8% within three years after a negative Pap in Ontario [7]. The purpose of our study was to explore the experiences of younger women with LACC requiring chemoradiation who underwent a Pap smear within two years of diagnosis. Specifically, we sought to explore experiences up-to and including their diagnosis (including cervical cancer screening), and how the diagnosis/treatment has changed their lives and outlook for the future. We hypothesized that women who underwent an unsuccessful Pap smear prior to their diagnosis would have strong opinions and views of cervical cancer screening and may provide insight into the care they received leading up to their diagnosis. Our objectives were to qualitatively study patients’ perceptions of presenting symptoms, experience with the health care system, feelings after diagnosis, and perception of their future after chemoradiation for LACC.

## Materials and methods

Our institution is a regional cancer hospital providing all radiotherapy required for a catchment population of more than 1.5 million. A search was performed of the electronic chart system to identify women diagnosed with cervix cancer, age below 50 at diagnosis, FIGO (International Federation of Gynecology and Obstetrics) Stage IB1 to IVA who received primary radiotherapy with concurrent chemotherapy with curative intent between September 2010 and December 2012. A priori, we decided to only include women under 50 as we were limited in our resources to interview women for this study and decided to focus our efforts on a select patient population. Participant interviews were arranged during routine follow up, with a paper and electronic chart review for documentation of previous Pap smears. All participants spoke adequate English, and interpreting services were not available for this project.

Participants were considered to be compliant with screening if in the preceding two years a participant underwent a Pap smear that did not warrant further attention, or if an abnormal finding on Pap smear was followed by colposcopy with no suspicion of cancer. Eligible participants were approached during a routine treatment/follow up visit to participate and provided with a letter of information. A single face-to-face interview was scheduled separate from routine clinical care by a gynecologic oncology disease site team social worker (HS) and research assistant (EJ) in an interview room in the clinic. The interview was conducted by HS with EJ aiding with transcription. Participants would have met HS if prior care with social work was provided. Adequate time (60-90 minutes) was allotted to explore the following areas in a semi-structured format: presenting symptoms; experience with the health care system; feelings after diagnosis; and perception of their future (see Appendix A for interview outline). Pilot testing was not performed. Detailed notes were taken, and the interview was audiotaped and transcribed. There was no further feedback sought from participants.

A thematic analysis was performed of the transcribed interviews using a stepwise inductive process [8]. The first step was familiarization of the interview data, which was accomplished through a single interviewer (EJ) transcribing the ten interview transcripts verbatim. Next, two additional co-investigators (DD, HS) independently read the interview transcripts and created an initial coding framework. These three co-investigators then met to discuss the framework and themes which emerged. Thematic analysis was performed using constant comparison to highlight differences and similarities in data between participants. Subsequently, the entire study team met to discuss the final study framework with the themes agreed upon by consensus and discussion of the interview transcripts where necessary (all authors). All authors approved the final manuscript. This research was approved by the Human Subjects Research Ethics Board at Western University, London, Ontario (REB# 103188).

## Results

Thirty-eight women were identified of which 13 met the eligibility criteria (38%). Ten women participated in an interview with their characteristics shown in Table 1. All were considered to be from an urban area. A normal Pap smear was done in the prior two years for all except one who was found to have LSIL (Low-grade squamous intraepithelial lesion) 11 months prior to diagnosis. Two participants had a normal Pap smear done in the context of prenatal care.

**Table 1:** Demographic characteristics of study participants FIGO: International Federation of Gynecology and Obstetrics

Characteristic	Number (Percentage)
Age	
20-29	2 (20%)
30-39	7 (70%)
40-49	1 (10%)
FIGO Stage	
1B1	3 (30%)
1B2	1 (10%)
2B	3 (30%)
3A	1 (10%)
3B	2 (20%)
Histology	
Squamous Cell Carcinoma	7 (70%)
Adenocarcinoma	3 (30%)
Primary Tumour Size	
< 4cm	2 (20%)
4-6cm	4 (40%)
> 6cm	3 (30%)
Unknown	1 (10%)
Smoking	
Yes	4 (40%)
No	6 (60%)
Diagnosis While Pregnant/Post-partum	
Yes	3 (30%)
No	7 (70%)
Has Children	
Yes	7 (70%)
No	3 (30%)
Has Partner	
Yes	6 (60%)
No	4 (40%)
Time between Diagnosis and Interview	
< 1 year	2 (20%)
1 – 2 years	4 (40%)
>2 years	4 (40%)

Six main themes were identified and are summarized in Table 2 along with associated subthemes and excerpts from research participants’ comments from the interviews:

**Table 2:** Themes identified using thematic analysis ER: emergency room; LACC: locally advanced cervical cancer; Pap: Papanicolaou.

Subthemes	Excerpts from research participants’ comments
Theme I: Belief that LACC does not occur in those who undergo screening	
Neither physician/patient considered cervix cancer because of Pap screening	“You’re too young and healthy to get cervix cancer”; “It took close to a year to be referred to a gynecologist but I think I was low priority because I had a normal Pap”; “When I asked my doctor if it could be cancer, he rolled his eyes and told me to stop looking on the Internet”
Normal Pap at beginning of pregnancy provided reassurance despite symptoms	“Although I was bleeding during my pregnancy, I was reassured by my doctor and the fact that my Pap was normal”; “The lower back pain and bleeding I was having was attributed to having a C-section or postpartum depression”; “With the spotting I was having while pregnant, a full exam was done. Although my cervix was described as friable, I was told nothing was wrong”
Theme II: Lack of understanding about LACC symptoms/diagnosis of cervix cancer	
Not aware of symptoms of cervix cancer	Symptoms were related to multiple factors including “stress”, “going off the Pill”, “pregnancy”; When seen in the ER…“The bleeding was attributed to my period and I was told to go home”
Did not know biopsy was needed as next step	“Since my Pap test was OK, I wasn’t sure what test needed to be done even though I knew something was wrong”; “Although I was seen for a biopsy within a month of referral, it was a year after my symptoms started”; “I wish I had been sent for colposcopy right away”
Initially didn’t understand seriousness of cancer	“I did not realize I was going to need this much treatment”; “My family did not recognize the seriousness of my disease”; “It was only when I was told I could not have surgery that I realized it was serious”
Theme III: Reluctance from health care providers to perform a pelvic examination in the presence of symptoms	
No pelvic exam done despite symptoms	“I was prescribed different oral contraceptives but not examined”; Several other tests done instead including “pregnancy test”, “ultrasound”, “MRI for back pain”; “…I had to lie about not having a family doctor and symptoms to get a referral”
Attribution of health concerns to psychological reasons	“I was told to stop worrying and even my husband thought I was crazy…”; “I was told everything was OK and I should avoid wearing tight jeans or using tampons”; “My doctor rolled his eyes and booked me to come back in 2 weeks”
Theme IV: Negative emotions including anger, shame, regret, mistrust	
Anger	“I was resentful having to spend time away from my children to get radiation”; “Society doesn’t understand cervix cancer like they do for breast cancer where there are fundraisers…”; “There was a weird feeling of vindication in proving that something was wrong”
Shame	“...why didn’t you go for your Pap test?”; “People associate cervix cancer with a certain lifestyle…”
Mistrust	“…little faith in the medical system when it has failed me”; “It’s hard to trust my doctor when they didn’t diagnose my cancer sooner”; “I don’t agree with the recommendation to decrease how often Pap tests are done”
Theme V: Changes in quality of life from treatment	
Physical	“My sex life isn’t what it was before”; “My whole life has changed with me no longer able to have children”; “…paid a huge price with menopause and I don’t want to take hormones”; “I always need to figure out where the bathroom is”
Psychosocial	“I feel anxious every time I come for a checkup about the cancer returning”; “…has changed my perspective on life”; “You find out who your real friends are”
Theme VI: Advice for other women	
Preventative Health	“I didn’t know that smoking was a risk factor for cervix cancer…”; “There’s a need for women to be educated about HPV and cervix cancer”; “Make sure you get your Pap” (disagreement on changing to q3 years)
Advocacy	“Do not take NO for an answer”; “Trust your gut feeling if you think something is wrong…”; “Ask for a second opinion if you are not comfortable with what you are being told”; “There needs to be a better understanding of the warning signs”

Theme I: Belief that LACC does not occur in screening compliant patients

With a prior unremarkable Pap smear, several participants expressed that they did not believe it was possible to have cervical cancer. Many felt a similar perception was imposed upon them by their healthcare providers, potentially leading theme III (reluctance from health care providers to perform a detailed pelvic examination in the presence of symptoms). One participant was told in the emergency room that vaginal bleeding was related to her menstrual cycle and sent home after a negative pregnancy test. Another was told she was “too young and healthy to have cancer”. Some described having their concerns dismissed when asking if they had cancer and being told to stop looking at the Internet. A Pap smear done as part of routine prenatal care may provide false reassurance even with symptoms. While bleeding during pregnancy was assessed, a diagnosis of cervix cancer was not considered initially. Another participant reported that lower back pain and bleeding was attributed to having a recent C-section and possibly postpartum depression.

Theme II: Lack of understanding about the symptoms/diagnosis of cervix cancer

Participants were not aware of the symptoms associated with cervix cancer and often related them to other factors such as stress or discontinuing an oral contraceptive. Similar examples related to health care providers attributing symptoms to pregnancy or menstruation. Furthermore, they were not certain how a diagnosis of cervix cancer is made and what additional tests they should ask for since a Pap smear was done and normal. With a lack of knowledge of these aspects, while some stated they knew something was “wrong”, they did not know how to proceed. If they had understood what was needed, they would have demanded a biopsy and been more persistent in advocating for their health. Even when a diagnosis was made, they did not envision the seriousness of the type/stage of cancer until they were advised to have radiation and chemotherapy instead of surgery.

Theme III: Reluctance from health care providers to perform a detailed pelvic examination in the presence of symptoms

A persistent theme was a reticence to perform a pelvic examination despite having associated symptoms such as pain and bleeding. One participant reported close to one-year elapsing between onset of symptoms and referral to a specialist; however, no pelvic exam was performed during this time. While health care providers sought to investigate the cause of symptoms, no pelvic exam was performed. Examples include pelvic ultrasound, magnetic resonance imaging (MRI) for back pain, and βHCG. There was a general perception of physician inattentiveness/dismissal of concerns with false reassurance given about the cause of bleeding. Some described feeling talked down to or not taken seriously. It is likely that in some cases an abnormal appearance of the cervix may have been overlooked, a view that some participants endorsed. One woman underwent trials of different oral contraceptives however a grossly abnormal cervix was evident by the time of referral. Another with persistent spotting during pregnancy was given reassurance and told she was unnecessarily worrying. She was diagnosed with LACC in her third trimester.

Theme IV: Negative emotions including anger, shame, regret, mistrust

Several negative emotions were associated with the diagnosis. Anger was expressed for a variety of reasons (the time taken to make a diagnosis, time away from family during treatment). Feelings of anger after diagnosis were also linked to that of vindication. In many cases they described persisting with complaints of something being wrong despite being told to the contrary. There was a bittersweet sense of redemption felt with a diagnosis made. A sense of shame was also expressed for several reasons. Some health care providers, friends, and family implied it was due to their stupidity that they didn’t participate in routine screening and ended up with LACC. One woman describes the diagnosis changing the way other people thought and felt about her and adversely affected the relationship with her best friend. In particular, her diagnosis was linked to a perceived lifestyle. Several participants expressed that cervix cancer is not viewed in the same way by society as a young woman diagnosed with breast cancer. Many expressed a sense of mistrust of the health care system and of physicians. They related it to a failure to diagnose a cancer they felt should be picked up by screening. Almost all the participants expressed feeling uncomfortable with recent recommendations to decrease the frequency of routine Pap smears, despite routine screening appearing to have failed them. One woman viewed the change in frequency of testing as a lack of respect towards women’s healthcare.

Theme V: Changes in quality of life from treatment

Several women felt that an earlier diagnosis would have meant they could have had surgery. Being told that their cancer was too advanced or better treated without surgery was viewed with disappointment. Permanent changes in quality of life from subsequent treatment included changes in sexual function and hormonal levels, bladder and bowel function and loss of fertility. This was viewed as having a negative effect on their quality of life, adversely affecting personal relationships and having a different perspective on life. Having missed the window of opportunity to have surgery was seen as having a “worse” cancer with a more adverse prognosis. There was also expressed a fear of having the cancer return particularly around the time of checkups.

Theme VI: Advice for other women

Most participants expressed regret that they did not advocate more strongly for themselves leading to a delay in a diagnosis being made. Participants encourage women to persist if something is wrong until a diagnosis is made and if necessary to seek additional opinions. They felt there is a role for educating women on HPV and cervix cancer including the signs and symptoms of cervical cancer and how a diagnosis should be made. While there was disagreement on the recommended frequency of Pap testing, they did feel women should be compliant with this screening method. Some have successfully quit smoking but were unaware it was a risk factor for cervix cancer.

## Discussion

This qualitative cohort study of young women treated for LACC confirms that a significant proportion (38%) developed disease despite receiving a Pap compliant with screening recommendations. Through semi-structured interviews, a number of themes are evident from their experience. Our study found that a significant minority of women who develop cervical cancer underwent Pap smears prior to their diagnosis.

The finding that women are unaware of the symptoms of cervix cancer in the era of routine Pap testing is not new. Few young females are aware of symptoms of cervix cancer, which could result in delays in diagnosis [9]. Delays in the diagnosis of cancer can be grouped into patient delays (i.e., delay to seeking medical attention) as well as provider delays (i.e., delay in making the diagnosis after first seeking medical attention). Not recognizing potential symptoms of cervix cancer was found to be a significant risk factor for patient delay in obtaining a cervical cancer diagnosis [10]. In our study, when women felt that something was wrong, they did not know how to proceed with making a diagnosis or advocate for themselves. While it is recognized by experts that no screening tool is perfect, our study would suggest that many patients may not recognize this.

Provider delays also exist in the diagnosis of cervical cancer, with issues including incomplete records at time of consultation at a sexual health clinic, no record of visualizing the cervix and in some cases no documentation of advice to re-attend the office for follow up [10]. Symptoms such as vaginal bleeding, including intermenstrual or postcoital, and vaginal discharge are more likely to be due to other factors such as contraceptive side effects or infection than cancer [11]. In our study, the possibility of cervix cancer was not initially considered either by the patient or the initial health care providers in several instances. There are a few possible reasons. With a lower incidence of cervix cancer in developed countries, LACC is relatively uncommon in the primary health care setting with 1550 estimated cases of cervix cancer in 2017 in Canada [12]. As a result, there may be unfamiliarity with the associated findings of cancer on speculum exam in the primary care setting. Similar to the perception by the public, with a recent normal Pap smear, there may be a cognitive bias against considering LACC by healthcare providers despite the presence of symptoms. This overlooks the fact that a Pap smear as a screening test does not perform well as a diagnostic testing for invasive cancer [13], and it is also possible to obtain a false-negative result [14]. Given a higher proportion of adenocarcinoma cases, it is possible that some cases elude routine screening without having surface involvement or being located higher in the cervical canal.

Statements made during the interviews support that women were falsely reassured by a normal Pap smear and thought it was not possible they could have cervix cancer. If this were recognized, women in our study stated they would have lobbied their health care team for further management and investigations. This line of thinking appears to be related to friends and family of those in our study, with women reporting feeling the assertion from those around them that the reason for developing cancer was non-compliance with screening or risky behaviours.

Interviewees noted a perceived reluctance to perform a pelvic examination at presentation. The best evaluation of the cervix is done by direct visualization and palpation; maneuvers which may have been omitted in some women prior to LACC diagnosis. A variety of reasons likely exist including limited training for primary care physicians, reluctance to perform a gynecologic exam in a young female and not appreciating the importance of a pelvic exam [15-16]. There appears to be a lack of knowledge in patients and society in general with regards to HPV, screening and cervix cancer, with women believing that a Pap smear is considered an omnipotent test where those compliant need to know nothing more [17-18]. This is not limited to cervical cancer, with young women having a poor understanding of breast cancer risk factors as well [19].

In our study, there was also a negative association made between cervix cancer and presumed sexual history. Participants dealt with a stigma and blame due to a presumed lack of compliance with screening resulting in additional challenges in dealing with a diagnosis of cancer. The long-term side effects from radiation with concurrent chemotherapy have significant impact in several domains of their health [20-21]. Many expressed that despite being diagnosed with cancer and needing multimodality treatment with long-term changes in quality of life, they felt isolated and ignored citing a lack of support groups, fundraising efforts and awareness about their disease compared to other cancers such as breast cancer.

There are both similarities and differences in our study compared to qualitative studies in women with breast cancer. In a Norwegian cohort of women who underwent a negative mammogram but soon after received a diagnosis of breast cancer (false negative), several women indicated they believed mammography would detect all breast cancers, similar to Theme I in our study [22]. However, in contrast to participants in our study, women who presented with symptoms (i.e., a palpable breast lump) still received a timely diagnosis even in the setting of a negative mammogram, which is in contrast to Theme II (Lack of understanding about LACC symptoms/diagnosis of cervix cancer) and Theme III (Reluctance from health care providers to perform a detailed pelvic examination in the presence of symptoms). We hypothesize this could be due to better breast cancer awareness (i.e., presenting with a lump in the breast is commonly recognized as potential breast cancer) versus more vague symptoms associated with LACC. There was also an element of distrust after a false negative mammogram, similar to Theme IV, and similar recommendations for women to undergo regular cancer screening even though the screening test had failed them (Theme VI) [22].

It is interesting that the decreased recommended frequency of Pap screening (to every three years) was viewed unfavorably by participants. If anything, it is the effectiveness of Pap screening that has likely led to complacency where a diagnosis of cervix cancer may be overlooked. Our findings do not suggest foregoing the recommendations for Pap screening but that there is a need to educate the general population that they do not provide immunity from developing cervix cancer.

In the developed world, HPV vaccination and HPV co-testing for cervical cancer screening are expected to further reduce the burden of cervical cancer. While follow-up on randomized trials of HPV vaccines have been relatively short, they appear to impart long-lasting immunity from HPV and are expected to decrease the number of cases of HPV related cancer [23-24]. In our jurisdiction, HPV screening and co-testing is not routine, however, it is expected to be more effective and likely cost-effective compared to Pap smears alone [25-26].

This study is limited as it represents findings from a single tertiary cancer care facility. However, patients came from multiple referring physicians over a large geographic area in a single-payer health care system. Our sample size was limited, making it possible that thematic saturation was not achieved. Resources for this study were limited and insufficient to interview additional participants, and therefore we chose a convenience sample of women under 50. The women in our study all received chemoradiation for their cervical cancer, and women who receive primary surgery may have different experiences than our cohort. While attempts were made to conduct the interview with open-ended questions, occasionally prompting was required (Appendix A) which may have led to bias in obtaining information. Our interview questions were not based on previous studies. Participants may have recall bias and selectively avoid certain aspects, particularly since eight of ten study participants were interviewed more than one year after their diagnosis. The Pap smear interval of two years was an accepted screening frequency at the outset of our study, but is now routinely recommended every three years [2]. This exploratory study did not look at more detailed demographic or disease information or review prior cytology/pathology that may provide additional insight.

The findings of our study may be addressed by education of women and their health care providers. Important messages from this study include considering cervix cancer in the differential diagnosis irrespective of screening status, the importance of a pelvic exam as part of routine care and particularly when there are symptoms.

## Conclusions

One-third of women presenting with LACC less than 50 years of age had appropriate Pap screening prior to diagnosis in this cohort and were diagnosed because they became symptomatic. Qualitative analysis identified several themes: I) Belief that LACC does not occur in those who undergo screening; II) Lack of understanding about LACC symptoms/diagnosis of cervix cancer; III) Reluctance from health care providers to perform a detailed pelvic examination in the presence of symptoms; IV) Negative emotions including anger, shame, regret, mistrust; V) Changes in quality of life from treatment; VI) Advice for other women. Providers and patients need to have an increased awareness of these symptoms even when Pap screening has occurred.

## Appendices

APPENDIX A

1. Tell me about your health experiences before your diagnosis. 

Prompts: What was your experience with prior screening? How often did you go to your doctor for physicals, how often did you have pap tests, do you have a consistent family physician or do you go to clinics/see residents/move frequently? Describe your relationship with your health care provider, ie how long have you known them, how often do you usually see them (Prior to cancer diagnosis), do you usually feel satisfied with how your concerns are addressed?

2. Tell me about your experiences leading up to diagnosis. 

Prompts: What symptoms did you have if any? When did they begin? At what point did you decide to seek medical attention (How long had you been experiencing the symptoms)? How did your health care provider address your symptoms? Did you feel listened to when you first discussed your symptoms? Did you feel dismissed? Did you feel like he/she took the necessary steps to investigate your symptoms? Was a second opinion an option? Would you have known how to seek further medical attention if your family physician did not refer you? How long after the symptoms began were you referred to a specialist (gynecologist or oncologist)? How many appointments did you have with your health care provider before a referral to a specialist was made?

3. Tell me about how you felt when you were diagnosed. 

Prompts: What were your thoughts when you were told you had cervix cancer? Shock? Surprise? Anger? How did you tell your family/friends about your diagnosis? What was their reaction(s)? 

4. Have these experiences affected how you see your future? Do you believe that the diagnosis process has had an impact on your prognosis? What, if anything, do you think should have gone differently during the process? What advice would you give a friend or family member who described concerning symptoms to you?

 ​​​​​
